# Laparoscopic cholecystectomy after gallbladder preservation: TG18 Delphi score quantifies surgical difficulty

**DOI:** 10.1186/s12893-026-03525-8

**Published:** 2026-01-26

**Authors:** Juxian Song, Qianlong Wu, Weikun Wu, Xing Wang, Hulin Wang

**Affiliations:** 1 Department of Hepatobiliary Surgery, The 925th Hospital of PLA Joint Logistic Support Force, Guiyang, 550009 China; 2 Department of Hepatobiliary Surgery, Affiliated Cancer Hospital of Guizhou Medical University, Guiyang, 550004 China; 3 General Surgery Department, The 925th Hospital of PLA Joint Logistic Support Force, Guiyang, 550009 China; 4 The 925th Hospital of PLA Joint Logistic Support Force, No. 67 Huanghe Road, Huaxi District, Guiyang, 550009 China

**Keywords:** Cholecystolithiasis, Choledochoscopic gallbladder-preserving surgery, Laparoscopic cholecystectomy, Surgical difficulty, Tokyo guidelines, Propensity-matched study

## Abstract

**Background:**

Despite cultural preferences for organ preservation in East Asia, choledochoscopic gallbladder-preserving surgery (CGPS) remains controversial given high recurrence rates. This study objectively quantified the increased technical complexity of laparoscopic cholecystectomy (LC) for recurrent cholecystolithiasis after prior CGPS using the Tokyo Guidelines 2018 (TG18).

**Methods:**

In this propensity-matched study (1:1, *n* = 220) conducted between 2020 and 2025, patients requiring LC due to recurrent cholecystolithiasis after prior CGPS were compared with patients with primary cholecystolithiasis undergoing LC. Investigators matched groups for TG18 severity grading, BMI, and biliary anomalies. Two blinded surgeons assessed the intraoperative findings using TG18 Delphi scoring (7-point scale). Primary outcomes included difficulty scores, critical view of safety (CVS) achievement, and bile duct injury.

**Results:**

The CGPS group demonstrated significantly higher median TG18 scores (34 [IQR 30–39] vs. 21 [18–24]; adjusted mean difference: Δ 14.0 points, 95% CI: 11.2–16.8; *p* < 0.001), primarily due to fibrotic adhesions: Calot’s triangle dense fibrosis (49.1% vs. 6.4%), partial scarring (21.8% vs. 0.9%), and diffuse scarring (15.5% vs. 0%; all *p* < 0.001). Surgeons achieved CVS less frequently in the CGPS group (83.6% vs. 98.2%, *p* < 0.001). TG18 scores > 25 predicted a fivefold increased conversion risk (aOR = 4.9, 95% CI: 2.3–10.6).

**Conclusions:**

Prior CGPS induces irreversible fibrosis that significantly increases reoperative difficulty (Δ 14 TG18 points), highlighting the need for careful patient selection in organ-preserving procedures. Definitive management with primary cholecystectomy remains the gold standard.

## Background

Cholecystolithiasis affects 10%–20% of adults globally, with international guidelines [1] establishing laparoscopic cholecystectomy (LC) as the definitive gold standard treatment. Despite prospective cohort data demonstrating 30%–50% 5-year recurrence rates [2], choledochoscopic gallbladder-preserving surgery (CGPS) persists as a culturally driven alternative in East Asia [3, 4]. This divergence stems from deeply held beliefs regarding organ integrity preservation, contradicting contemporary recommendations: the Chinese Society of Surgery explicitly contraindicates CGPS due to recurrence risks [5], while endoscopic specialists permit selective application for patients with strong preservation preferences [6].

Dense fibrotic adhesions complicate secondary LC for recurrent cholecystolithiasis after prior CGPS, obscuring critical anatomy in 62%–78% of cases [2, 7]. This reoperative challenge lacks objective assessment tools, relying on subjective intraoperative judgments. Although the Tokyo Guidelines 2018 (TG18) Delphi criteria provide validated difficulty scoring for acute cholecystitis [8], their application in recurrent cholecystolithiasis after prior CGPS remains unexplored—a critical gap in surgical safety quantification.

To address this specific unmet need in surgical complexity stratification following gallbladder preservation, we implemented the internationally validated TG18 Delphi system [9] within propensity-matched case‒control cohorts comparing secondary LC for recurrent cholecystolithiasis after prior CGPS versus patients with primary cholecystolithiasis undergoing LC. We hypothesized that prior CGPS would yield a clinically significant increase in TG18 difficulty scores (Δ ≥ 12 points), with fibrosis-associated intraoperative findings contributing dominantly to score variance, and that scores exceeding the 25-point threshold would predict a fivefold elevation in conversion risk. This study establishes the first evidence-based stratification system for surgical complexity in secondary cholecystectomy following gallbladder preservation.

## Methods

### Study design and setting

This single-center retrospective case‒control study was conducted in the Hepatobiliary Surgery Department at the 925th Hospital of the Joint Logistics Support Force between January 2020 and June 2025. The case group (CGPS group) included patients requiring LC for recurrent cholecystolithiasis after prior CGPS, while the control group (primary LC group) comprised primary cholecystolithiasis patients undergoing LC without any history of gallbladder intervention. Investigators used propensity score matching (1:1 ratio) to control for potential confounders, including acute cholecystitis severity graded according to the TG18 criteria [10], body mass index (BMI), and biliary anomalies. All operative records and video archives were stored on secured, nonnetworked hospital servers. Two blinded senior hepatobiliary surgeons independently performed TG18 Delphi scoring. All LCs were performed by a dedicated hepatobiliary surgery team. The procedures were directly performed or supervised by a senior attending surgeon with experience of > 1000 LCs, ensuring a consistent surgical standard across both groups. The Institutional Review Board approved the study protocol (No. 925HL-2025-011), which complied with the Declaration of Helsinki (2013 revision) and STROBE guidelines; written informed consent was obtained preoperatively from all participants.

### Participants

Consecutive patients with radiologically confirmed cholecystolithiasis were enrolled, including the CGPS group and the primary LC group. All patients met surgical indications per the Chinese Expert Consensus on Surgical Management of Benign Gallbladder Diseases (2021 Edition) for elective or emergency LC [5]. The exclusion criteria were as follows: [1] postoperative gallbladder malignancy; [2] non-CGPS upper abdominal surgery; [3] prior percutaneous transhepatic cholangiodrainage (PTCD)—excluded due to peritoneal adhesions altering surgical anatomy [10]; and [4] cirrhosis. We verified CGPS cases through operative reports and registry audits.

### CGPS procedural specifications

All index CGPS procedures strictly adhered to the Clinical Guideline for Choledochoscopic Gallbladder-Preserving Surgery (2021 edition) [6]. Preoperative evaluation mandated comprehensive assessment, including hepatobiliary ultrasound and magnetic resonance cholangiopancreatography (MRCP), to evaluate gallbladder morphology, wall integrity, mucosal status, and stone characteristics. Functional assessment comprised lipid-stimulated ultrasound for gallbladder contraction studies and quantitative measurement of gallbladder concentration capacity using ^99m^Tc-ethylfeninate hepatobiliary scintigraphy (ECT). Eligible patients presenting with cholecystolithiasis were classified according to the Tokyo Guidelines 2013 (TG13) criteria, which are equivalent to the TG18 severity grades: [1] asymptomatic chronic cholecystitis; [2] TG13 Grade I (mild) acute calculous cholecystitis; or [3] TG13 Grade II (moderate) acute calculous cholecystitis that had resolved following at least 3 months of conservative management.

Two principal surgical approaches were utilized: The percutaneous technique involved a mini-laparotomy (≤ 5 cm) in the right upper quadrant with a direct fundal incision for choledochoscope access. The laparoscopic approach, defined by all procedural steps performed entirely under laparoscopic visualization, was classified into three variants: [1] multiport laparoscopy [2], single-incision laparoscopy, and [3] needlescopic-assisted laparoscopy, based on the number, size, and location of abdominal trocar sites.

The core surgical sequence involved creating gallbladder access through the selected approach, achieving complete clearance of gallbladder stones under direct choledochoscopic visualization, and selectively resecting localized pathological mucosa when indicated. Following meticulous inspection, surgeons closed the gallbladder in two layers using absorbable sutures. Given the elective nature and the resolved inflammatory state of the gallbladder in all included cases, no cholecystostomy tubes were placed during the index CGPS procedures. Selective placement of cholecystostomy tubes and/or abdominal drains preceded standard fascial and cutaneous closure.

### Variables and data sources

The primary exposure variable was intraoperative difficulty assessed using the 25-item Delphi scoring system (7-point scale per item: 0 = least to 6 = most difficult, validated by Asia-Pacific consensus) [9]. Both assessors were experienced hepatobiliary surgeons (each having performed > 1000 laparoscopic cholecystectomies) who were blinded to the patient group assignment. They completed calibration using the standardized TG18 typical difficulty video library prior to independent scoring to ensure consistency [11]. In addition to the total score, the highest single-item score for each procedure was identified. Based on this highest item score, the overall surgical difficulty was classified into one of five levels (Level 1 to Level 5), as recommended by the TG18 group for clinical interpretation [12]. Primary outcomes included achievement of the Critical View of Safety (CVS) assessed according to Strasberg criteria (i.e., complete dissection of Calot’s triangle, separation of the gallbladder from the cystic plate, and visualization of only two structures entering the gallbladder) [13] and incidence of bile duct injury [14]. Documented surgical characteristics encompassed approach (standard, lateral dorsal infundibular approach [15], subtotal cholecystectomy, fundus-first dissection, or conversion to open surgery) and infundibular management (routine clipping, fenestration, or reconstitution) [8]. Perioperative metrics comprised operative duration (minutes), intraoperative blood loss (ml), postoperative bile leakage (defined as > 50 ml/24 h from drains or radiologically confirmed intra-abdominal collection), and length of postoperative hospitalization (days). For patients in the CGPS group, the interval between the prior gallbladder-preserving procedure and the subsequent laparoscopic cholecystectomy was calculated. This interval was defined as the time in months from the date of the index CGPS to the date of the current LC performed for recurrence. Confounding factors included acute cholecystitis severity (assessed using the TG18 severity grading, which maintains the same criteria as TG13), BMI (pathological obesity ≥ 30 kg/m²), biliary anomalies per Blumgart’s system [16] (including anomalous biliary duct unions > 2 cm from hilar confluence and accessory ducts) and accessory ducts. Data acquisition involved blinded assessment of surgical videos and operative records for Delphi scoring. Researchers extracted perioperative variables from anesthesia records, nursing charts, and electronic medical records.

#### Bias

To address inherent cohort imbalance (approximately 20 CGPS group patients versus 200 primary LC group patients annually), propensity score matching (1:1 ratio with nearest-neighbor algorithm and 0.2 caliper width) adjusted for confounders associated with surgical difficulty: TG18 severity grading (criteria consistent with TG13), BMI, and biliary anomalies. Standardized mean differences (SMD) < 0.1 across all matched covariates confirmed adequate intergroup balance postmatching [17].

#### Study size

A priori power analysis (G*Power 3.1; α = 0.05, β = 0.2, effect size d = 0.8 based on pilot data indicating a 12-point TG18 score difference) determined a minimum requirement of 77 patients per group, accounting for 15% anticipated attrition. Among 1,273 consecutive patients with radiologically confirmed cholecystolithiasis treated between January 2020 and June 2025, we identified 118 eligible CGPS group patients and 823 eligible primary LC group patients. Propensity score matching yielded 110 covariate-balanced pairs (93.2% matching efficiency), achieving > 99% power to detect the prespecified effect size (d = 0.8) postmatching.

### Quantitative variables

Laboratory parameters for TG18 severity grading were dichotomized per guideline thresholds: white blood cell count > 18 × 10⁹/L, platelet count < 100 × 10⁹/L, international normalized ratio (INR) > 1.5, serum creatinine > 176.8 µmol/L, and PaO₂/FiO₂ ratio < 300 mmHg. All other continuous variables (including operative duration and blood loss) retained continuous-scale values.

### Statistical methods

We performed propensity score matching using SPSSAU^®^ (v24.0; QingSi Technology) with the nearest-neighbor algorithm (1:1 ratio, 0.2 caliper width). Consistent with contemporary recommendations [16], we intentionally omitted postmatching hypothesis testing for baseline covariates to prevent alpha-error inflation; covariate balance evaluation relied exclusively on SMD, as detailed in the Bias section. All statistical analyses were performed using SPSS version 25.0 (IBM Corp.). Normality was assessed by the Shapiro‒Wilk test. Categorical variables are presented as frequencies (%), with between-group comparisons by *χ*² or Fisher exact tests. Continuous variables are expressed as the mean ± SD (normal distribution) or median (IQR) (nonnormal distribution) and were analyzed via paired t tests or Wilcoxon signed-rank tests. Interrater reliability for TG18 items was quantified using Cohen’s κ coefficient, with weighted κ for multilevel classifications. Statistical significance was defined as two-tailed *p* < 0.05.

## Results

### Participants and descriptive data

**Fig. 1:**
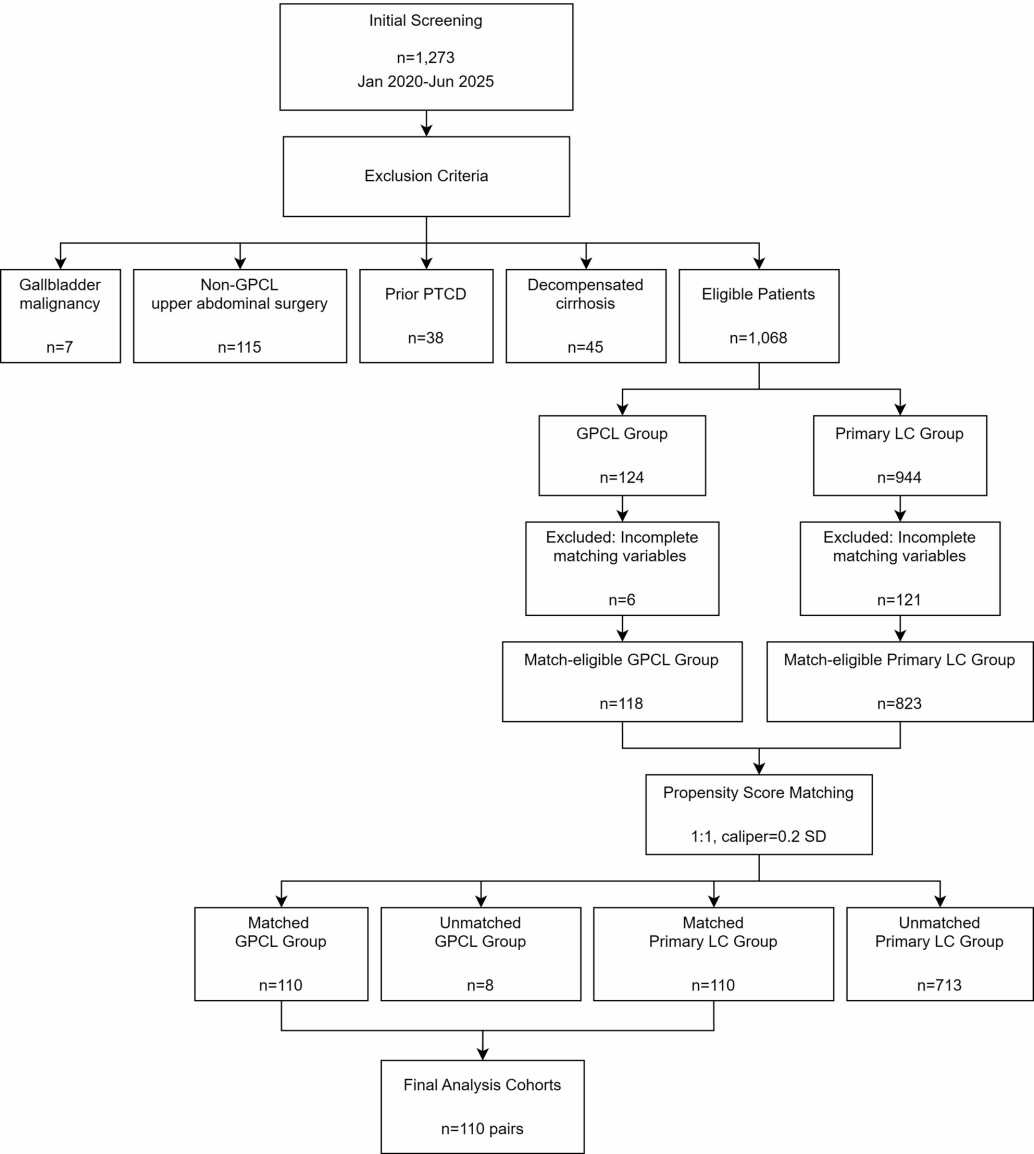
Patient Screening and Propensity Matching Workflow. This flowchart illustrates the sequential process of patient identification, inclusion/exclusion criteria application, and propensity score matching

After screening 1,273 patients who underwent LC, we excluded 205 based on predefined criteria: postoperative gallbladder malignancy (*n* = 7), history of non-CGPS upper abdominal surgery (*n* = 115), prior PTCD (*n* = 38), and decompensated cirrhosis (*n* = 45). The final analytical cohort comprised 1,068 patients: 124 in the CGPS group and 944 in the primary LC group. After excluding 6 patients from the CGPS group and 121 patients from the primary LC group due to missing propensity matching variables (TG18 severity grading, BMI, biliary anomalies), 118 patients in the CGPS group and 823 patients in the primary LC group remained for matching. Propensity score matching (1:1 ratio, caliper width = 0.2 SD) yielded 110 matched pairs, with 8 unmatched patients in the CGPS group (93.2% matching efficiency). All SMDs for matched covariates were < 0.1 (Fig. 1; Table 1). Complete intraoperative documentation and follow-up data were available for all 220 matched patients.

**Table 1:** Baseline characteristics before and after propensity score matching

Characteristic	Before Matching		SMD	After Matching		SMD
	**CGPS** **Group** (*n* = 118)	**Primary LC Group** (*n* = 823)		**CGPS Group** (*n* = 110)	**Primary LC Group** (*n* = 110)	
**Age**,** years**	54.2 ± 12.8	48.7 ± 14.3	0.32	53.9 ± 11.6	54.1 ± 12.1	0.03
**Male sex**,** n (%)**	68 (57.6)	389 (47.3)	0.18	63 (57.3)	64 (58.2)	0.01
**TG18 severity grade**,** n (%)**						
Grade I	18 (15.3)	312 (37.9)	0.41	17 (15.5)	18 (16.4)	0.05
Grade II	76 (64.4)	398 (48.4)	0.28	71 (64.5)	70 (63.6)	0.03
Grade III	24 (20.3)	113 (13.7)	0.53	22 (20.0)	22 (20.0)	0.07
**BMI**,** kg/m²**	26.8 ± 3.5	23.1 ± 2.9	0.67	26.5 ± 3.2	26.3 ± 3.0	0.08
**Biliary anomalies**,** n (%)**	21 (17.8)	98 (11.9)	0.31	19 (17.3)	**16 (14.5)**	**0.10**
**Diabetes history**,** n (%)**	32 (27.1)	172 (20.9)	0.22	30 (27.3)	31 (28.2)	0.04
**WBC count**,** ×10⁹/L**	12.3 ± 4.2	11.8 ± 3.7	0.15	12.1 ± 3.9	12.0 ± 4.0	0.06

Notes: Data presented as the mean ± standard deviation or n (%). SMD: Standardized mean difference; values < 0.1 indicate adequate balance. Matching variables: *TG18* Severity grade, *BMI* Biliary anomalies. Bold values indicate key modifications

Among the matched CGPS patients, the interval to reoperation exhibited a right-skewed distribution, with a median of 22.5 months (14.0–38.0) and a range from 4 months to 9 years, indicating that most recurrences clustered within the first 3 years. When stratified by the median interval, TG18 difficulty scores were comparably elevated in both the early (≤ 22.5 months, *n* = 55; median score 35 [31–40]) and late (> 22.5 months, *n* = 55; median score 34 [30–39]) subgroups compared to the primary LC group (median score 21 [18–24]; both *p* < 0.001). No significant correlation was found between the continuous interval length and TG18 total score (Spearman’s *ρ* = 0.08, *p* = 0.41).

### Outcome data

Among matched patients in the CGPS group (*n* = 110), index gallbladder-preserving procedures comprised percutaneous CGPS (*n* = 17) and laparoscopic CGPS (*n* = 93). Surgeons achieved CVS significantly less frequently in the CGPS group (83.6%, 92/110) than in the primary LC group (98.2%, 108/110; *p* < 0.001). All 18 CVS failures in the CGPS group resulted from fibrotic adhesions in Calot’s triangle, categorized as dense fibrosis (*n* = 9), partial scarring (*n* = 6), or diffuse scarring (*n* = 3). No Strasberg grade B/C bile duct injuries occurred in either cohort. Table 2 presents distributions of surgical approaches (standard, lateral dorsal infundibular approach, subtotal cholecystectomy, fundus-first dissection, conversion) and infundibular management strategies (routine clipping, fenestration, reconstitution).

**Table 2:** Surgical approaches and cystic infundibulum management strategies in matched cohorts

Variable	CGPS (*n* = 110)	Primary LC (*n* = 110)	*P* value
**Surgical approach**			< 0.001
Standard	51 (46.4%)	84 (76.4%)	
Lateral dorsal infundibular approach	36 (32.7%)	17 (15.5%)	
Subtotal cholecystectomy	5 (4.5%)	3 (2.7%)	
Fundus-first	15 (13.6%)	6 (5.5%)	
Conversion to open surgery	3 (2.7%)	0 (0.0%)	
**Cystic infundibulum management**			0.039
Clipping	102 (92.7%)	104 (94.5%)	
Fenestration	1 (0.9%)	1 (0.9%)	
Reconstruction	7 (6.4%)	5 (4.5%)	

Note: Data are presented as the number of patients (percentage). *P* values were calculated using Fisher's exact test for categorical variable distributions. “Reconstruction” refers to the suture closure of the residual cystic infundibulum after clipping or division, performed to reduce the risk of bile leakage. These distributions reflect intraoperative adaptations to prior CGPS fibrotic adhesions, although surgical strategy selection remains operator dependent. The findings should be interpreted as descriptive trends rather than definitive qualitative evidence due to potential confounding by surgeon experience and preference

### Main results

As detailed in Table 3, the CGPS group exhibited significantly elevated TG18 Delphi difficulty scores compared to the primary LC group (adjusted mean difference: 14.0 points, 95% CI: 11.2–16.8; *p* < 0.001), exceeding the predefined clinical significance threshold (Δ ≥ 12 points). Application of the TG18 difficulty classification revealed a profound shift in the case-mix complexity, with a significantly greater proportion of high-difficulty procedures (Level 4 or 5) in the CGPS group (69.1% vs. 0.0%; *p* < 0.001) and nearly one-quarter (21.8%) categorized as Level 5 exclusively in this group (Table 3). Interrater reliability for adhesion assessment showed excellent agreement (weighted κ = 0.86, 95% CI: 0.79–0.93), and the consistency between assessors for the total TG18 score was also excellent, with an intraclass correlation coefficient (ICC) of 0.91 (95% CI: 0.87–0.94). Pericholecystic adhesion analysis revealed higher rates of partial scarring in the CGPS group (27.3% vs. 1.8%; *p* = 0.006), with suture-induced diffuse scarring exclusively in 9 cases in the CGPS group (8.2%). Calot’s triangle evaluation demonstrated significantly more severe fibrotic alterations in the CGPS group: dense fibrosis without scarring (49.1% vs. 6.4%; *p* < 0.001), partial scarring (21.8% vs. 0.9%; *p* < 0.001), and diffuse scarring (15.5% vs. 0%; *p* < 0.001). While gallbladder bed morphology did not differ significantly (*p* = 0.32), noninflammatory adhesions (33.6% vs. 10.0%; *p* = 0.003) and gallbladder neck mounting on the common bile duct (18.2% vs. 2.7%; *p* = 0.002) were more prevalent in the CGPS group. Crucially, TG18 scores > 25 predicted conversion risk (adjusted OR = 4.9, 95% CI: 2.3–10.6; *p* < 0.001), confirming the hypothesized fivefold increase.

**Table 3:** TG18 Delphi difficulty scores and intraoperative findings

Variable & TG18 item	CGPS Group (*n* = 110)	Primary LC Group (*n* = 110)	*P* value
Pericholecystic appearance			
Partial scar adhesions (Item 2)	30 (27.3%)	2 (1.8%)	0.006*
Diffuse scarring (Item 3)	9 (8.2%)	0 (0.0%)	< 0.001*
**Calot’s triangle assessment**			
Dense fibrosis without scars (Item 5)	54 (49.1%)	7 (6.4%)	< 0.001*
Partial scarring (Item 6)	24 (21.8%)	1 (0.9%)	< 0.001*
Diffuse scarring (Item 7)	17 (15.5%)	0 (0.0%)	< 0.001*
**Intra-abdominal factors**			
Noninflammatory adhesions (Item 23)	37 (33.6%)	11 (10.0%)	0.003*
GB neck mounting on CBD (Item 25)	20 (18.2%)	3 (2.7%)	0.002*
**Highest difficulty level**,** n (%)**			< 0.001*
Level 1	0 (0.0)	15 (13.6)	
Level 2	3 (2.7)	68 (61.8)	
Level 3	31 (28.2)	27 (24.5)	
Level 4	52 (47.3)	0 (0.0)	
Level 5	24 (21.8)	0 (0.0)	
Level 4 or 5 (High Difficulty)	76 (69.1)	0 (0.0)	
**Global difficulty score**			
Median (IQR)	34 (30–39)	21 (18–24)	< 0.001‡
Adjusted mean difference (95% CI)	14.0 (11.2–16.8)	-	< 0.001*

Note: Data presented as n (%) or median (interquartile range). *P values from chi-square test; ‡*P* value from Mann‒Whitney U test. Adjusted mean difference calculated via linear regression controlling for TG13 severity grade. A TG18 score > 25 predicted conversion risk (adjusted OR = 4.9, 95% CI: 2.3–10.6; *p* < 0.001). Interrater reliability: weighted κ = 0.86 (95% CI: 0.79–0.93). CBD: common bile duct

### Other analyses

The median total postoperative hospital stay did not differ significantly between the CGPS group and the primary LC group (5 days [4–7] vs. 4 days [3–6]; *p* = 0.152). Clavien‒Dindo grade I-II complications occurred significantly more frequently in the CGPS group (18.2% vs. 8.2%; *p* = 0.029), including postoperative paralytic ileus (*n* = 2), hospital-acquired pneumonia (*n* = 4), acute decompensated heart failure (*n* = 1), and catheter-associated urinary tract infections (*n* = 3). Bile leakage rates did not differ significantly (CGPS group: 0.9% [1/110] vs. primary LC group: 1.8% [2/110]; *p* = 0.621). No 30-day mortality or in-hospital deaths occurred. Subgroup analyses by prior CGPS approach (percutaneous vs. laparoscopic) showed no significant differences.

## Discussion

Standardized TG18 assessment objectively demonstrates that prior CGPS induces irreversible fibrotic adhesions within Calot’s triangle, substantially elevating anatomical complexity during subsequent LC. It is important to note that our study employed a retrospective design, applying the TG18 grading system to a mix of surgical videos and detailed narrative records. While assessors were calibrated using standard video libraries to ensure fidelity to the TG18 criteria, the inherent heterogeneity in documentation may introduce variability not present in prospective studies that use exclusively standardized video footage. This represents both a pragmatic adaptation of the scoring system and a limitation in the context of perfect measurement reproducibility. Notwithstanding this methodological consideration, the objective data consistently demonstrate that prior CGPS induces irreversible fibrotic adhesions within Calot’s triangle, which substantially elevates anatomical complexity during subsequent LC. This anatomical restructuring compounds technical obstacles in achieving CVS and heightens risks of anatomical misinterpretation [3, 14]. Crucially, although our cohort showed no statistically significant increase in major complications despite elevated difficulty, this outcome reflects specialized expertise—hepatobiliary specialists performed all procedures on high-risk referrals, excluding extreme-risk populations (e.g., cirrhotic patients). Within broader healthcare ecosystems, particularly resource-constrained settings, these cases present considerable hazards: surgeons encountering fibrotic “frozen triangles” risk catastrophic consequences from subtle anatomical errors, even when open conversion prevents bile duct injury [18]. Consequently, LC represents restricted salvage therapy for recurrent cholecystolithiasis after CGPS rather than universally applicable management [19, 20]. Furthermore, the increased operative difficulty was independent of the timing of reoperation. In our cohort, the median interval from CGPS to LC was approximately 1.9 years, and stratification analysis confirmed that TG18 scores remained comparably and markedly elevated both within the first 2 years and beyond. This pattern suggests that the fibrotic remodeling induced by CGPS constitutes a persistent and irreversible anatomical challenge. This stands in sharp contrast to the situation following percutaneous drainage for acute cholecystitis, where a deliberate delay to allow inflammation to subside is often beneficial and recommended [21].

Methodological limitations necessitate cautious interpretation of risk profiles. Single-center outcomes from senior surgeons cannot be extrapolated to diverse practice environments [22]. First, excluded populations with portal hypertension (e.g., cirrhotic/PTCD patients) likely exhibit adhesive patterns exceeding the predictive capacity of current scoring systems [23]. Second, the reliance of Delphi scoring on expert-reviewed documentation cannot address decision-making in settings lacking video recording capabilities—a common scenario in community hospitals [24]. Third, absent data on independent procedures by junior surgeons obscures real-world safety profiles beyond supervised academic settings [25]. While these design elements strengthen internal validity, they inadvertently mask the full risk spectrum of reoperation after CGPS.

These findings require integration into Chinese surgical policy frameworks. Aligning with the Chinese Society of Surgery consensus prioritizing index cholecystectomy [5], our data substantiate restrictive indications for gallbladder-preserving strategies. Although proponents emphasize patient autonomy and organ conservation [26], the exponential increase in reoperative difficulty fundamentally challenges long-term safety—particularly given that ~ 70% of biliary operations in China occur outside tertiary centers. The absence of bile duct injuries in our series paradoxically underscores the steep learning curve of the technique: safety is achievable solely through stringent case selection and centralized expert performance. International multicenter data reporting 1.2%–3.8% bile duct injury rates after CGPS likely reflect generalized realities more accurately [27, 28]. Thus, the primary contribution of this study lies in quantifying how CGPS generates complex surgical sequelae that may outweigh transient benefits, not in affirming reoperation safety.

Clinical implementation demands tiered risk stratification. Chronic fibrosis after CGPS creates irreversible anatomical distortion distinct from acute inflammation where conservative management permits tissue recovery. CGPS-induced scarring forms anatomical labyrinths necessitating strict resource allocation when reoperation is unavoidable: Community hospitals should classify LC after CGPS as restricted technology—our data show 16.4% CVS failure rates even in optimized cohorts (vs. 1.8% in primary cholecystolithiasis), a threshold predisposing to injury with inexperienced operators [29]. Regional hepatobiliary centers must establish credentialing requiring ≥ 50 ACC Grade III operations [30] with mandatory multidisciplinary consultation for intraoperative scores breaching critical thresholds. Informed consent documents should explicitly state: “Gallbladder preservation multiplies reoperative difficulty” and “Community facilities lack resources for managing such cases” [31]. This stratified approach mitigates medico-legal risks in biliary surgery while upholding *primum non nocere*.

## Conclusions

The TG18 Delphi scoring system objectively quantifies significantly increased anatomical complexity during LC after prior CGPS, driven by irreversible fibrotic remodeling. While specialized centers can reduce major complications, these findings support restrictive patient selection for gallbladder preservation within the tiered healthcare system of China, requiring centralized management of reoperations to counterbalance inherent technical hazards.

## Data Availability

The datasets used and/or analyzed during the current study are available from the corresponding author upon reasonable request.

## Abbreviations

CGPS: Choledochoscopic Gallbladder-Preserving Surgery
CVS: Critical View of Safety
ECT: ^99m^Tc-Ethylfeninate Hepatobiliary Scintigraphy
INR: International Normalized Ratio
LC: Laparoscopic Cholecystectomy
MRCP: Magnetic Resonance Cholangiopancreatography
PTCD: Percutaneous Transhepatic Cholangiodrainage
SMD: Standardized Mean Differences
TG13: Tokyo Guidelines 2013
TG18: Tokyo Guidelines 2018
